# Sediment Bacterial Communities Reflect the History of a Sea Basin

**DOI:** 10.1371/journal.pone.0054326

**Published:** 2013-01-23

**Authors:** Christina Lyra, Hanna Sinkko, Matias Rantanen, Lars Paulin, Aarno Kotilainen

**Affiliations:** 1 Department of Food and Environmental Sciences, University of Helsinki, Helsinki, Finland; 2 Institute of Biotechnology, University of Helsinki, Helsinki, Finland; 3 Geological Survey of Finland, Espoo, Finland; Utrecht University, The Netherlands

## Abstract

How entire microbial communities are structured across stratified sediments from the historical standpoint is unknown. The Baltic Sea is an ideal research object for historical reconstruction, since it has experienced many fresh- and brackish water periods and is depleted of dissolved oxygen, which increases the sediment's preservation potential. We investigated the bacterial communities, chemical elements (e.g. Cr, Pb Na, P, Sr and U) and sediment composition in a stratified sediment core dated by radiocarbon and spanning 8000 years of Baltic Sea history, using up-to-date multivariate statistics. The communities were analysed by 16S rRNA gene terminal restriction fragment length polymorphism. The communities of the deep Early Litorina and surface Late Litorina Sea laminae were separated from the communities of the middle Litorina Sea laminae, which were associated with elevated concentrations of U and Sr trace elements, palaeo-oxygen and palaeosalinity proxies. Thus, the Litorina Sea laminae were characterized by past oxygen deficiency and salinity increase. The communities of the laminae, bioturbated and homogeneous sediments were differentiated, based on the same historical sea phases, with correct classifications of 90%. Palaeosalinity was one of the major parameters that separated the bacterial communities of the stratified sediments. A discontinuous spatial structure with a surprising increase in community heterogeneity was detected in Litorina Sea sediments from 388 to 422 cm deep, which suggests that a salinity maximum occurred in the central Gulf of Finland app. 6200–6600 years ago. The community heterogeneity decreased from the surface down to 306 cm, which reflected downcore mineralization. The plateau of the decrease was in the app. 2000-year-old sediment layers. Bacterial community data may be used as an additional tool in ocean-drilling projects, in which it is important to detect mineralization plateaus both to determine historically comparable portions of sediment samples and historical events, such as sea-level rise culminations.

## Introduction

Despite ongoing ocean and continental drilling programmes, which investigate the history of sea basins, there is a lack of theory on how entire microbial communities are structured across stratified sediments. Generally, microbial species richness and biodiversity are expected to decrease with depth [1]. Wu et al. [2], however, detected increased species richness and biodiversity in their deepest sediment layers. It is not known whether the associations between the habitats and microbial communities simply reflect downcore mineralization shifts or also changes in deposition history and, thus, the history of a sea basin.

The Baltic Sea is an ideal research object for studying the history of a sea basin, since it has been influenced by land uplift and sea-level rises (transgression), resulting in many fresh- and brackish water phases [3], [4]. The Early Litorina Sea, a transition between the freshwater Ancylus Lake and the brackish Litorina Sea stage, may have ended app. 8500–7400 calibrated years before the present (cal years BP) [3], [5]. The entire Litorina Sea phase with the sea-level rise culmination (transgression maximum) and the salinity maximum, followed by the present Late Litorina Sea, may have ended app. 4000–3000 cal years BP [3].

Inflows of saline waters through the Danish Straits most probably caused the development of a halocline limiting the vertical water circulation in the Litorina sea-level rise culmination [6]. The halocline resulted in oxygen depletion in the bottom sediments, slowing down the mineralization of the organic matter deposited [7]. The transgression and salinity maxima occurred in distinct areas of the Baltic Sea at different times; e.g. high salinities occurred in the Baltic Proper and Northern Baltic Sea, app. 6000–5000 [8], [9] or 6700–6400 cal years BP [10], respectively. We do not, however, have detailed information on the palaeosalinities of the central Gulf of Finland (GOF).

The Baltic Sea phases were previously determined, based on proxies such as diatoms, stable isotopes (e.g. ^13^C∶^12^C ratio of total organic carbon (TOC) and Sr isotopes) and lithostratigraphical features (e.g. organic-rich laminae) [5], [6], [8], [9], [11], [12]. Low oxygen conditions at the seafloor and consequently lack of sediment reworking by benthic animals (bioturbation), increase preservation of the laminated structures in sediments [13]. Laminae, in turn, in oxic conditions are reworked more homogenous by bioturbation. Sr isotopes were recently used as palaeosalinity proxies in the Baltic Sea [9]. In addition, elevated concentrations of trace elements, such as U and Sr, are considered as signs of palaeosalinity increases and palaeo-oxygen decreases [6], [11]. For instance, U precipitates onto surfaces of depositing particles in oxygen-depleted conditions [14]. To date, the best method form confirming the salinity changes between freshwater and brackish water is the diatom record [12]. However, diatom preservation declines in many different environmental states, such as bioturbation and coarseness of sediment [15]. Thus, additional palaeo-environmental tools are needed.

Although ancient [16], [17] microbes belonging to palaeome [18] and present-day [19], [20] sediment microbial communities have been analysed, using various molecular biomarkers, Mantel correlograms, have not been used previously to investigate how entire bacterial communities are structured in stratified sediments. Multivariate statistics has likewise not been used to study combined microbial, chemical and geological datasets from the palaeo-environmental standpoint.

We investigated whether the salinity changes in the Baltic Sea basin can be inferred from bacterial community data. We analysed the associations between bacterial community composition and the spatial, sedimentological as well as chemical element parameters and radiocarbon dating in stratified Baltic Sea sediments from the central GOF, spanning a historical period of 8000 years. Using multivariate methods, such as piecewise Mantel correlograms, generalized discriminant analysis and canonical analysis of principal coordinates (CAP), we showed that the past salinity changes and sudden slowdown of organic material mineralization in the Baltic Sea basin, as well as downcore mineralization, can be inferred from the entire body of bacterial community data.

## Results

### Chemical-bacterial community composition interactions

**Figure 1:**
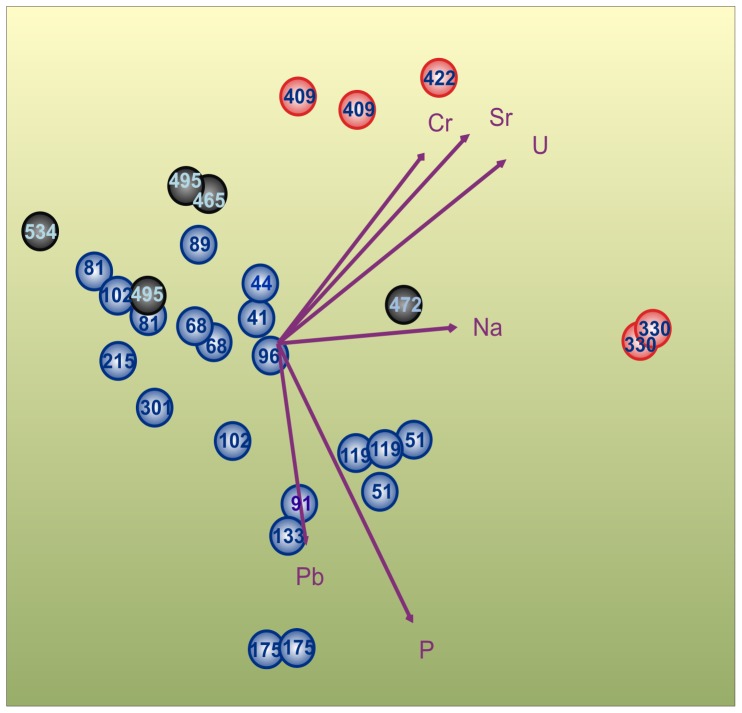
Association between bacterial sediment communities and chemical variables in Baltic Sea laminae. The black-filled circles indicate the sediment samples from the Early Litorina, red from the Litorina and blue from the Late Litorina sea phases. The numbers are rounded to the nearest integer. Canonical analysis of principal coordinates was based on the Bray Curtis dissimilarity matrix (n = 32×158) of bacterial terminal restriction fragments of the 16S rRNA gene. Samples and chemical parameters (purple arrows) were plotted against canonical axis scores 1 and 2. The chemical variables chosen explained 45% of the variation. Test statistics with 9999 permutations resulted in a highly significant *p* value (*p* = 1e-04), which allows rejection of the null hypothesis that there are no relationships between the bacterial communities and chemical variables. The length of the arrow indicates the strength of the correlation between the sediment samples and chemical parameter. An arrow direction indicates the increasing concentration of the chemical parameter.

CAP analysis of bacterial 16S rRNA gene terminal restriction fragment (T-RF, Table S1) and chemical element data, such as Cr, Pb, Na, P, Sr and U concentrations (Table S2), derived from organic-rich laminae (Figure S1) showed that the bacterial communities of the deep Early Litorina laminae could be grouped with the communities of the surficial Late Litorina Sea laminae (Figure 1). The communities of the Litorina layers in the middle were associated with elevated concentrations of trace elements, particularly with U and Sr, and formed a separate group.

Cr, Pb, Na, P, Sr and U variables were most significantly associated with T-RFs (Figure S2 and Table S3) and were therefore chosen to the final model (Figure 1). Most of the chemical variables (such as Al, Ba, Ca, C, Co, Fe, Mg, N, Tl and V) were collinear, highly depth-dependent (Table S4) and showed a clear non-linear relationship with T-RFs (Figures S3 and S4) and, thus, were not included in the final model.

Variance partitioning determined that chemical parameters explained 37.7%, whereas spatial parameters explained only 4.9% of the variation in the bacterial community data of the laminae. Therefore, the chemical parameters, which reflect the quality of organic matter, best explained the changes in bacterial community compositions in the dataset.

### History of a sea basin as revealed by bacterial community composition

**Figure 2:**
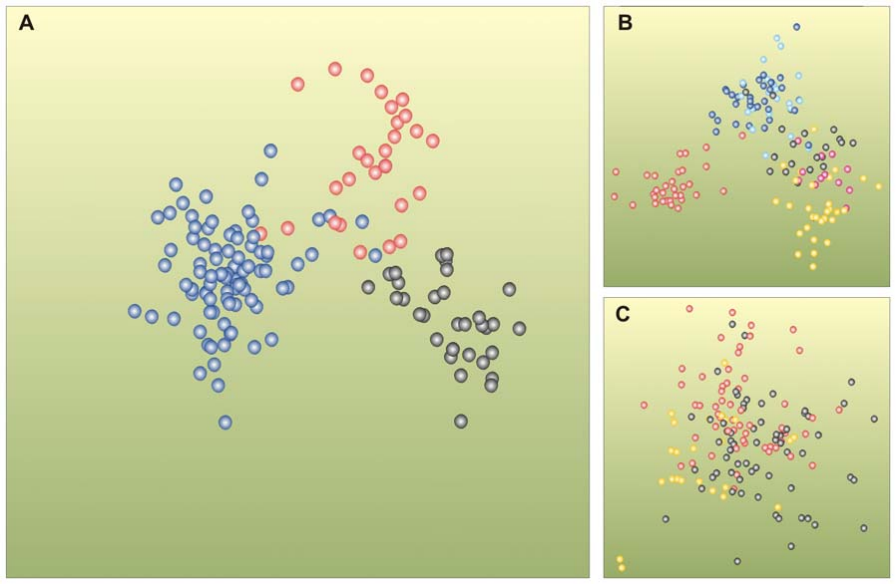
Differentiation of Baltic Sea bacterial communities based on sea phases, depth and sediment composition. (A) Shea phases: black filled circles indicate sediment samples from the Early Litorina, red from the Litorina and blue from the Late Litorina sea phases. (B) Depth classes: pink-filled circles indicate sediment samples from 533.5 to 500.75 cm, yellow from 494.85 to 403.75 cm, black from 393.7 to 301.35 cm, turquoise from 297.5 to 203.50 cm, blue from 199 to 101 cm and red from 96 to 18.25 cm below the seafloor. (C) Sediment composition: black-filled circles indicate laminae, yellow bioturbated and red homogeneous sediment layers. Generalized discriminant analysis was based on Bray and Curtis distances of bacterial terminal restriction fragments of the 16S rRNA gene (n = 148×199). Correct classification was a) 90.5%, b) 78% and c) 52.7%. Test statistics with 9999 permutations resulted in a highly significant *p* value for a) and b) (*p* = 1e-04) which allows rejection of the null hypothesis of no differences among the *a priori* groups. The default option of canonical analysis of principal coordinates, which combines unconstrained and constrained multivariate methods, resulted in a) 24, b) 23 and c) 13 principal component axes that were further analyzed by generalized discriminant analysis. The first and second canonical axes were plotted here.

General discriminant analysis of *a priori* groups (Table S5) of bacterial communities in the sediment core sample differentiated the communities, based on the past sea phases: Early Litorina Sea, Litorina Sea and Late Litorina Sea (Figure 2A). The bacterial communities formed groups representing presumed sea phases, with correct classification of 90% (Figure 2A). The communities were grouped also based on various depth classes, however, with a lower correct classification of 78% (Figure 2B). The bacterial communities were not, however, clearly separated, based on sedimentological parameters, laminae, bioturbated or homogeneous sediments (Figure 2C).

### Spatial structure of the bacterial communities in stratified sediments

**Figure 3:**
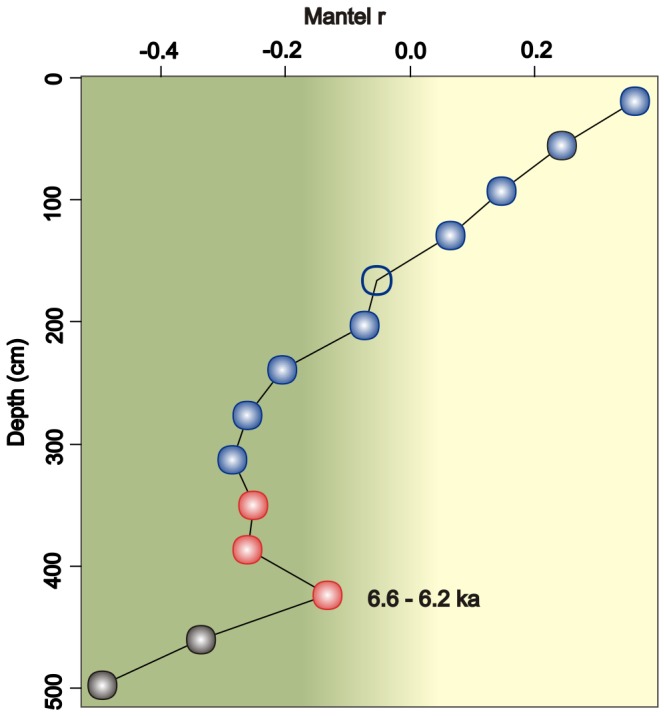
Spatial structure of bacterial communities along a sediment sample covering 8000 years of Baltic Sea history. The piecewise Mantel correlogram was based on the Bray and Curtis dissimilarity matrix (n = 148×219) of bacterial terminal restriction fragments of the 16S rRNA genes. Autocorrelations (Mantel r) were plotted against distance classes (Table S6), which were based on the Euclidean distance between sampling depths. Red, green and black circles represent the distinct trends detected. Filled circle: *P*<0.05. Open circle: nonsignificant value.

Piecewise Mantel correlograms revealed a discontinuous spatial structure in the bacterial communities (Figure 3, Table S6) in stratified sediments (Figure S1). The long-term linear drifts from 18 down to 306 cm (Figure 3) illustrated the decrease in heterogeneity (increase in entropy) of the bacterial community with depth. The plateau of the decrease in heterogeneity was at 306 cm, in the app. 4500-year-old sediment layers (Figure 3). Surprisingly, the increase in heterogeneity of the bacterial community composition was detected from 388 down to 422 cm in the app. 6200–6600-year-old sediment layers (Figure 3).

### Methane signature bacteria

**Figure 4:**
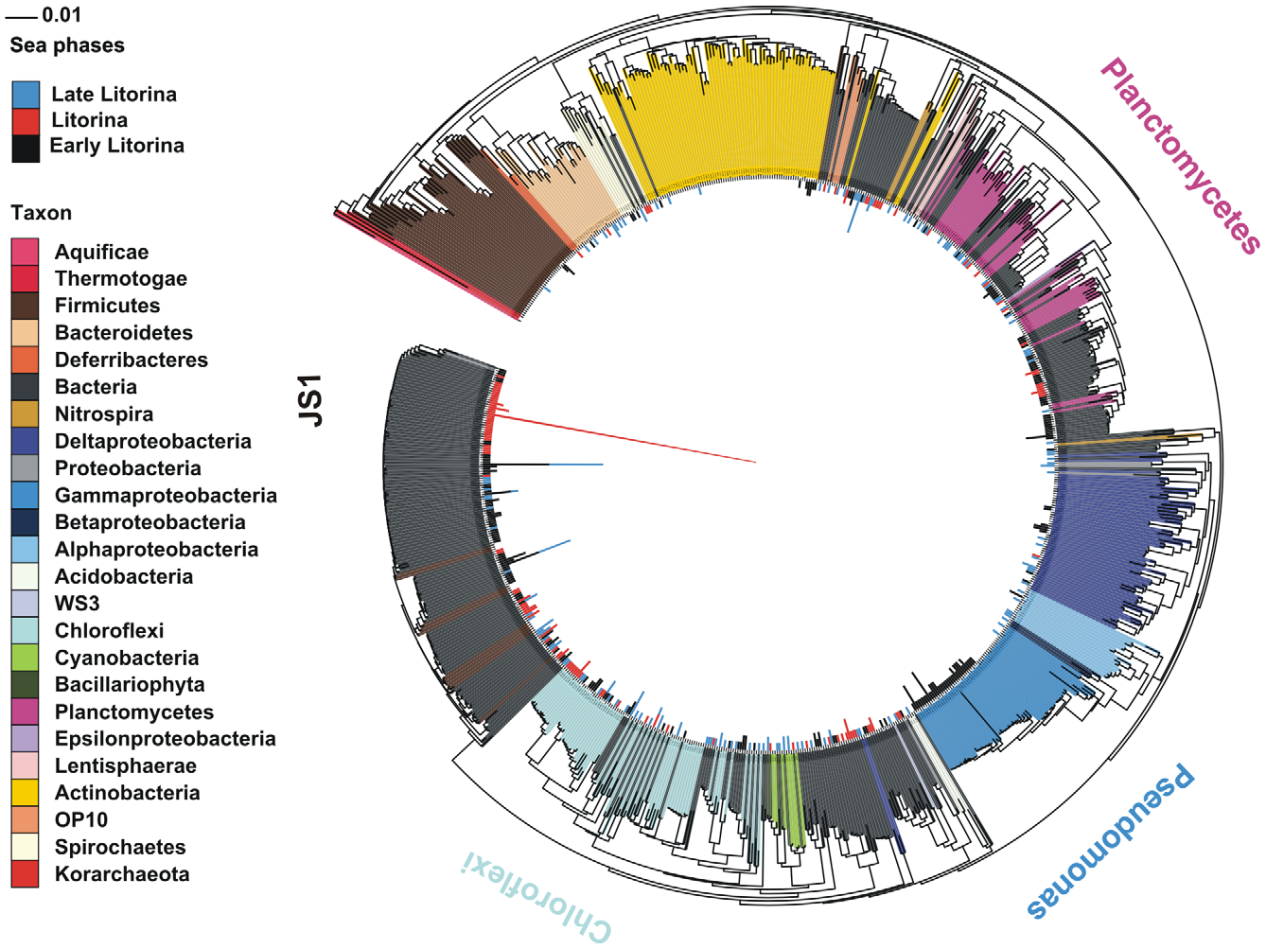
Neighbor-joining phylogenetic tree of the partial 16S rRNA genes from stratified Baltic Sea sediments. In the inverted circular tree with a stacked bar chart (sea phases), red bars indicate the abundance of a leaf (sequence) found in the Litorina Sea laminae (depths 330 and 422 cm), blue bars the abundance of a leaf found in the Late Litorina Sea laminae (depths 91 and 101 cm) and the black bars the abundance of a leaf found in the Early Litorina lamina (depth 534 cm). The leaves without bars are reference sequences obtained using the NAST tool. Reliable branches are shown in the bootstrap tree (Fig. S5). The clone sequences were assigned to phylum or class level (Proteobacteria) or to genus level (*Pseudomonas*) by the RDP classifier with an 80% threshold. The putative JS1 clones were assigned, based on the closest sequence matches, using the RDP seqmatch tool. Scale bar represents the genetic distance, i. e. the nucleotide substitutions per site.

Methane hydrate signatures, such as those of *Chloroflexi*, *Planctomycetes*, and putative candidate division bacteria JS1 were abundant in clone libraries from all sea phases (Figures 4 and S5), based on assignment of the sequences with a naïve Bayesian classifier and the seqmatch tool of the Ribosomal Database Project (RDP). JS1 candidate division bacteria were found throughout the sediment core in high abundances (observed T-RFs 221 and 223 in Table S1). Both the clone libraries and the T-RFs elucidated that JS1 bacteria predominated in the Baltic Sea subsurface sediment. JS1 bacteria may be the key players in organic- and methane-rich brackish subsurface sediments.

Certain JS1 16S rRNA gene sequences formed a microdiverse gene cluster, that was found only in the Litorina Sea layers (Figures 4 and S5). The separate gene cluster included a high number of a particular JS1 phylotype in the saline Litorina layer (high red bar in Figures 4 and S5). This suggests that the abundant phylotype in the cluster is or has become evolutionarily well adapted to contemporary or past environments.

### Signals from times gone by

In contrast to the adapted microdiverse phylotype of JS1, *Pseudomonas* bacteria seemed to emerge suddenly in an 8000-year-old (7600 cal years BP) Early Litorina layer (Figure 4) The middle Litorina Sea phase was characterized by non-nitrogen-fixing *Synechococcus* (T-RFs 126, 128, 136, 137 and 139 in Figure S6), whereas the younger Late Litorina Sea phase in turn was characterized by filamentous nitrogen-fixing cyanobacteria, such as *Nodularia* (fragments 289, 291 and 293 in Figure S6). The majority (66–68%) of the sequences in each clone library remained unidentified, which indicates that the bacteria belong to previously undescribed phyla.

## Discussion

CAP analysis of the bacterial communities of the laminae spanning the 8000-year history of the Baltic Sea showed that the communities of the deep Early Litorina Sea and the surficial Late Litorina Sea laminae were grouped together. The Litorina Sea laminae in the middle formed a distinct group. The communities in the Litorina Sea laminae were associated with elevated concentrations of U and Sr. The elevated concentrations of trace elements, such as U, in these layers suggest that they are characteristic of past oxygen deficiency [6]. The trace element U is also considered as a sign of palaeosalinity increase [11], together with Sr. The result suggests that the bacterial communities of the Litorina Sea laminae reflect past salinity increase, which affected halocline formation, causing oxygen deficiency [6].

The Early Litorina Sea, the transition phase between the Ancylus lake and Litorina Sea, may have ended app. 8500–7400 cal years BP [3], [5] whereas the entire Litorina Sea phase with the salinity maximum, followed by the Late Litorina Sea, may have ended app. 4000–3000 cal years BP [3]. Based on generalized discriminant analysis, the bacterial communities of the laminae, bioturbated and homogeneous sediments formed three distinct groups representing the presumed Early Litorina Sea, Litorina Sea and Late Litorina Sea periods, with correct classification of 90%. Therefore, we concluded that palaeosalinity is probably one of the major parameters that affected bacterial communities in the sediment core. Thus, our bacterial data show strong evidence supporting the sea transitions of the Baltic Sea.

To date, the best way to confirm the salinity changes between freshwater and brackish water is the diatom record [12]. In lake sediments, diatom preservation declines with increasing pH, temperature, grazing, bioturbation and coarseness of sediment [15]. Here, we suggest that bacterial community composition data could be used as an additional tool in palaeosalinity estimations, especially if diatom records have not been preserved.

The bacterial communities in the laminated sediments were not separated from the communities of the bioturbated or homogeneous sediments. A previous parallel DNA and lipid biomarker analysis showed that the biomarkers were either present or absent in a particular sediment layer [21], which suggests that DNA does not migrate vertically. Thus, the most probable explanation for our result is that the quality and chemical composition of the organic matter, as well as the environmental conditions, are the same in the laminated and bioturbated sediments within a sea phase. Therefore, the bacterial community composition was not changed, due to the rough sediment composition characteristics.

The bacterial communities of the stratified sediments became structured discontinuously with depth. The bacterial heterogeneity decreased in the surficial sediment layers with depth and reached the plateau in the app. 4500-year-old layers, but suddenly increased in the app. 6200–6600-year-old sediment layers. The gradual heterogeneity loss in the bacterial communities was presumable, since it is governed by the exhaustion of reactive organic matter in the sediment with time. However, the surprising increase in heterogeneity may reflect the changes in the organic matter deposited and thus past climatic and oceanographic periods of the Baltic Sea.

The increase in heterogeneity of the bacterial community composition may be explained by the preserving nature of the sediment layers. The high preservation potential of the Litorina Sea sediment was due to an inflow of saline water through the Danish Straits, resulting in a halocline formation [6]. Consequently, oxygen deficiency slowed down the mineralization of organic matter [7]. The Litorina transgression maximum, occurred in the eastern GOF between app. 7400–7100 cal years BP and 6500–6300 cal years BP [22], whereas on the southern coast of the GOF the transgression maximum occurred earlier: between 7600–7300 and 6900–6500 cal years BP [23], which could represent the maximum sea surface salinity in the region [23].

However, so far we have no detailed information on salinity changes in the study area, the central GOF. In our study, the increased heterogeneity of the bacterial community composition record suggests that the high salinity app. 6600–6200 years ago influenced the past environments and thus the composition of the material deposited in the central GOF.

The layers with high preservation potential may support organic matter- decomposing bacterial communities and shelter organic matter, such as DNA itself. A large part of the sedimentary DNA may be extracellular and the mineralization of DNA low in anoxic organic-rich sediments [24]. The sedimentary microbial activities in the core studied here may be very slow and the community structure may not have changed to a large extent since the original deposition. We concluded that at least below the plateau of mineralization, the layers were comparable from the palaeo-environmental standpoint.

The 16S rRNA gene sequences of *Chloroflexi*, *Planctomycetes* and particularly the putative candidate division bacteria JS1 were abundant in clone libraries from all the sea phases. The abundance of *Chloroflexi*, *Planctomycetes* and JS1 methane process signatures [25], [26] in the Baltic Sea sediment sample core may have been due to a diffuse methane flux from buried organic material boosting an ongoing biogenic anaerobic oxidation of methane (AOM). AOM was previously linked with a diffuse methane flux from brackish laminated subsurface sediments [20].

A microdiverse 16S rRNA gene cluster of certain JS1 bacteria emerged in the Litorina Sea layers. A changed environment causes adaptive evolutionary branching, which can be classified as a separate DNA sequence cluster without even knowing the ecology of the organism [27]. In our study, the adapted ecotype of JS1 in the saline Litorina Sea layers was seen as a separate microdiverse gene cluster, including a high number of a particular phylotype in the Litorina layer. This suggests that the phylotype predominating in the cluster is or has become evolutionarily well adapted to contemporary or past environments. Coolen et al. [17] previously detected change in a JS1 phylotype along different sediment units. These JS1 biomarkers could be used as tools to synchronize the sediment core records sampled worldwide.

The presence of *Pseudomonas* bacteria, which seemed to emerge in the old Early Litorina sediment layer, may be explained by the strong input of terrestrial organic matter 8000 years ago, although contamination cannot completely be ruled out. An inflow of nutrient-rich marine water through the Danish Straits, may have boosted cyanobacterial primary production during the Litorina transgression [11], [28]. Indeed, our study showed that non-nitrogen-fixing unicellular *Synechococcus* was a typical taxon emerging during the Litorina Sea phase. The combined pigment and nitrogen data suggested that filamentous nitrogen-fixing cyanobacteria were also important primary producers during the Litorina transgression [29]. However, based on our results filamentous nitrogen-fixing cyanobacteria, such as *Nodularia*, were characteristic of the younger Late Litorina Sea. Filamentous nitrogen-fixing cyanobacteria may have become common more recently, in the brackish Late Litorina Sea.

The majority (66–68%) of the sequences in each clone library remained undescribed and were classified only at the domain level of bacteria. This stresses how little we still know about subsurface bacterial communities.

## Conclusions

Multivariate methods, such as piecewise Mantel correlograms, have not previously been used to investigate the spatial structure of bacterial communities in downcore studies. Seldom, if ever, have the combined datasets of microbial and geological data been studied with multivariate statistics from the palaeoenvironmental standpoint. We showed that entire bacterial communities reflect both ancient and contemporary events, such as salinity changes and ongoing mineralization processes, using multivariate statistic and phylogenetics. Piecewise Mantel correlograms detect mineralization plateaus and thus determine the historically comparable portions of vertical sediment samples. The method may also reveal palaeoenvironmental events, such as relative sea-level rise culminations (sea transgressions). These tools may be useful in ocean-drilling projects, which that examine the history of the earth recorded beneath the seafloor.

## Materials and Methods

### Sediment sampling

The sediment core was recovered, using a piston corer from the Gulf of Finland (GOF) site GF2 (Lat 59°50.38′N, Long 25°51.86′E; water depth 84 m) during a SEQUE1 2004 cruise on the r/v Aranda in April 2004. The sediment core was stored at an *in situ* temperature (4°C) until subsampling. The subsamples were taken for terminal-restriction fragment length polymorphism (T-RFLP) and cloning analysis and stored at −20°C and at −70°C until used. No specific permits were required for the field study described. The location is not privately owned or protected. The field study did not involve endangered or protected species.

### Sediment composition, chemical and dating data

The sediment core was photographed and sedimentological descriptions, e.g. organic-rich laminae and signs of bioturbation in the sediment (bioturbated homogeneous sediment) of the core were done from the split and trimmed sediment surface. Geochemical analyses of parallel sediment samples were performed at the analytical chemistry laboratory of Labtium Oy (Espoo, Finland). The sediment samples were freeze-dried, sieved to obtain the <2-mm fraction, homogenized and digested, using hydrofluoric acid and perchloric acid. The samples were analysed for carbon and nitrogen, using a Leco carbon-hydrogen-nitrogen CHN-600 analyser (Leco Corp., St. Joseph MI, USA) while phosphorus and 29 other element concentrations (Table S2) were obtained with inductively coupled plasma-mass spectrometry (ICP-MS) and inductively coupled plasma-atomic emission spectrometry (ICP-AES), respectively.

The sediment samples were dated, using the radiocarbon accelerator mass spectrometry (AMS-^14^C) method. The radiocarbon AMS-^14^C analyses were done at the radiocarbon Dating Laboratory of Lund University in Sweden. The AMS-^14^C dates were calibrated to calendar years (0 cal. Before the Present (BP) = 1950), using CALIB REV 6.0.1 software. The values were corrected for the reservoir effect by applying the Marine04 calibration dataset with the Baltic Sea regional average ΔR value of −107±24 [30].

### DNA extraction and amplification of the 16S rRNA gene

DNA was extracted from app. 0.25 g of the sediment sample, using a Power Soil DNA extraction kit (MoBio Laboratories Inc., Carlsbad, CA, USA). The bacterial 16S rRNA gene was amplified in triplicate for T-RFLP analysis and for constructing clone libraries. The primer 27f with and without FAM (5′ terminus labelled with 6-carboxyfluorescein and 1405r (acgggcggtgtgta) (Oligomer Oy, Helsinki, Finland), modified from 1406r [31] was used in a PCR for T-RFLP and cloning.

Amplification was done in a total volume of 25 µl of 1× DyNAzyme reaction buffer (Finnzymes Oy, Espoo, Finland) with 0.2 µM of both primers, 0.2 mM of dNTP (Finnzymes Oy), 0.15 mM MgCl_2_ (Finnzymes Oy), DyNAzyme™ II DNA polymerase 2U (Finnzymes Oy) and app. 3 µl of DNA. The amplification protocol was performed at 94°C for 3 min, 35× (at 94°C for 30 s, 52°C for 30 s and 72°C for 1 min) and at 72°C for 10 min. The triplicate products were pooled and purified, using an EZNA Cycle Pure kit (Omega Bio-Tek Inc., Norcross, GA, USA) for both T-RFLP and cloning.

### Terminal-restriction fragment length polymorphism

Two hundred ng of the amplification products were digested with 5 U of the restriction enzyme HaeIII (several products were also cut separately with HhaI, MspI and RsaI, Promega Corp., Madison, WI, USA) in reaction buffer C (Promega) for 4 h at 37°C. One µl of the restriction digestion product was mixed with 0.2 µl of MapMarker® 1000 (Bioventures Inc., Murfreesboro, TN, USA) internal size standard and 20 µl of HiDi formamide (Applied Biosystems, Foster City, CA, USA) and denatured at 95°C for 5 min. The T-RFs were separated with the polymer POP7 and a 3130×l Genetic Analyzer (Applied Biosystems) at 60°C with 15 kV for 30 min.

The T-RFs were determined by their sizes with Peak Scanner™ Software (Applied Biosystems), using the Local Southern method. All T-RFs over baseline fluorescence units and with lengths from 50 to 500 bp were rounded to the nearest integer and normalized with Initiative for Bioinformatics and Evolutionary Studies (IBEST) tools [32]. True peaks (operational taxonomic units, OTUs) were distinguished from background ‘noise’, based on three-fold standard deviation (IBEST default). Comparable OTUs from the samples were binned with IBEST tools to decrease bias in fragment-size determination. The relative abundances of the binned fragments (Table S1) were used in statistical analysis.

### 16S rRNA gene cloning and sequencing

The 16S rRNA genes were cloned with the TOPO TA Cloning® kit (Invitrogen, Carlsbad, CA, USA). The colony-PCR was done in a total volume of 25 µl of 1× DyNAzyme reaction buffer consisting of 0.5 µM of M13f and M13r primers (Sigma-Aldrich, St. Louis, MO, USA), 50 µM of dNTP (Finnzymes Oy), 0.15 mM MgCl_2_ (Finnzymes Oy) and DyNAzyme™ II DNA polymerase 1.2 U (Finnzymes Oy). The amplification protocol was performed at 94°C for 10 min, 35× (at 94°C for 30 s, 56°C for 30 s and 72°C for 30 s) and at 72°C for 10 min. The 16S rRNA genes were sequenced with pD′, using the BigDye terminator method. The sequenced 16S rRNA genes were identified with a naïve Bayesian classifier (Release 10.10) of the Ribosomal Database Project (RDP) [33] and based on the closest sequence matches, using the RDP seqmatch tool, version 3 [34]. Sequence data have been deposited under accession numbers FR819782–FR820455 in EMBL.

### Identification of terminal-restriction fragments

The T-RFs were identified, based on virtually (*in silico*)-digested and identified 16S rRNA gene clones, using the Bioperl tool T-DistinctiEnz (Bioinformatics Organization; http://www.bioinformatics.org/~docreza/rest_html/home.htm). The *in vitro* T-RF was considered as identified if a comparable T-RF were found with four different enzymes in the *in silico* digestion with a shift of ±2 bp between the *in vitro* and *in silico* T-RFs.

### Statistical analyses

To investigate the relationships between the bacterial T-RFs and chemical parameters, the significant chemical variables (P<0.05) were preselected (Tables S2 and S3), using marginal tests of multivariate multiple regression, the DISTML*forward* program [35], [36]. The significant variables were used as explanatory variables in the initial model of Canonical Analysis of Principal Coordinates CAP [37].

Most of the chemical variables showed a non linear relationship with the bacterial communities when fitted on the bacterial ordination surface (Figures S3 and S4) and were highly depth-dependent (Table S4). To determine the chemical and bacterial associations, the most explaining (Table S3), non collinear (Table S4) and certain non linear chemical variables (Figure S2) were chosen for the final linear model presented here. CAP was performed, using the R package Vegan [38] functions ‘capscale’ for CAP, ‘ordisurf’ for fitting the chemical parameters on the ordination of bacterial communities and ‘permutest’ for testing the significance.

To analyse the spatial structure of the bacterial (T-RFs) data with depth (Euclidean distance), a piecewise Mantel correlogram using the R package Ecodist function ‘pmgram’ [39] was performed in an R environment [40]. Default options were used to determine the number and width of the distant classes (Table S6). Generalized discriminant analysis [41], using the R package BiodiversityR [42] functions ‘CAPdiscrim’ for discriminant analysis and ‘permutations’ for testing the significance, was done to test the difference between the *a priori* groups of multivariate observations (Table S5). The *a priori* groups of the bacterial communities (Table S5) were formed, based on the Mantel correlogram results (Figure 3, Table S6), depth and rough sediment composition (laminae, bioturbated homogeneous and homogeneous sediment). The homogeneity of the group variances (Figure S7) was analysed by a multivariate analogue of Levene's test, an analysis of multivariate homogeneity of group dispersions [43], using the R package Vegan [38] functions ‘betadisper’ for group dispersions and ‘permutest’ for testing the significance.

Variance in the bacterial communities was partitioned in to pure chemical and depth proportions [35], [44]–[47]. The first partial RDA runs were done, using the program DISTML [48], and were subsequently used in variance partitioning. In all statistical analyses, the Bray-Curtis distances were calculated between observations and 9999 permutations were used for calculating the *P* values.

### Phylogenetics

The closest uncultured and cultured sequence matches were determined using the Nucleic Acid Simulation Tool (NAST) (http://greengenes.lbl.gov/) [49] and aligned with our sequences using my RDP [34]. The neighbor-joining tree was constructed with the phylogeny Inference Package (PHYLIP) [50], using the ‘seqboot’, ‘dnadist’, ‘neighbor’ and the ‘consense’ programs. The seqboot bootstrapping tool was used for generating multiple datasets (1000), which were used as input data in the downstream analysis.

The distance matrix was computed (‘dnadist’) with multiple datasets under the nucleotide substitution model F84. The neighbor-joining tree was constructed, using the ‘neighbor’ program. Multiple tree files, derived from dnadist, were run by the ‘consense’ program, resulting in a majority rule consensus tree. The majority rule phylogenetic tree was drawn by the Interactive Tree of Life web service (http://itol.embl.de/) [51].

## Supporting Information

Figure S1 **Sediment core taken from the northern Baltic Sea.** Two homogeneous mud units contain both homogeneous and bioturbated layers.
(PDF)

Figure S2 **Relationship between chromium, lead, sodium, phosphorus, strontium, uranium and bacterial communities.** The chemical variables were fitted on the final bacterial ordination surface. The numbers in the coloured filled circles indicate the subsurface depth of a particular sample (bacterial communities determined by T-RFLP). The green numbers indicate the concentration of a particular chemical parameter. Final model: refer to Figure 1.
(PDF)

Figure S3 **Non linear relationship between aluminium, barium, calcium, carbon, cobalt, iron and bacterial communities.** The chemical variables were fitted on the final bacterial ordination surface. The numbers in the coloured filled circles indicate the subsurface depth of a particular sample (the bacterial communities determined by T-RFLP). The green numbers indicate the concentration of a particular chemical parameter. Final model: refer to Figure 1.
(PDF)

Figure S4 **Non linear relationship between magnesium, nitrogen, thallium, vanadium and bacterial communities.** Chemical variables were fitted on the final bacterial ordination surface. The numbers on the coloured filled circles indicate the subsurface depth of a particular sample (the bacterial communities determined by T-RFLP). The green numbers indicate the concentration of a particular chemical parameter. Final model: refer to Figure 1.
(PDF)

Figure S5 **Bootstrap tree of the partial 16S rRNA genes from stratified Baltic Sea sediments.** In the inverted circular tree with a stacked bar chart, the red bars indicate the abundance of a leaf (sequence) found in the Litorina Sea laminae (depths 330 and 422 cm), blue bars the abundance of a leaf found in the Late Litorina Sea laminae (depths 91 and 101 cm) and black bars the abundance of a leaf found in the Early Litorina lamina (depth 534 cm). The leaves without bars are reference sequences obtained, using the NAST tool. The clone sequences were assigned to phylum or class level (Proteobacteria) or to genus level (*Pseudomonas*) by the RDP classifier with an 80% threshold. The putative JS1 clones were assigned, based on the closest sequence matches, using the RDP seqmatch tool. The tree is based on 1000 bootstraps. Those branches with higher than 80% bootstrap values are indicated with red (maximum values) and green (minimum values) colour.
(TIF)

Figure S6 **Association between bacterial communities and terminal-restriction fragments from Baltic Sea sediments.** The expected/observed (larger font and dark grey) terminal-restriction fragments (T-RFs) of JS1 are 223–225/221, 223; *Synechococcus*: 128/126, 128 and 137/136, 137, 139; *Nodularia*: 291/289, 291, 293. The T-RFs expected were determined based on *in silico*-digested 16S rRNA gene clones. The bacterial communities were determined, based on HaeIII digested 16S rRNA gene clones. A shift of ±2 between the *in vitro* and *in silico* T-RFs was allowed. Samples (n = 148) are indicated with the blue (Late Litorina Sea)-, red (Litorina Sea)- and black (Early Litorina Sea)- filled circles. The numbers represent (in bp) (T-RFs, n = 219). Only T-RFs with canonical scores above ±1 for axis 1 and 2 were included. The clone sequences were identified, based on the closest sequence matches, using the RDP seqmatch tool.
(PDF)

Figure S7 **Variance heterogeneities of the 16S rRNA gene terminal-restriction fragment data.** (A) Sea phase, (B) depth classes and (C) sediment composition data were calculated by analysis of multivariate homogeneity of group variances (multivariate analogue of Levene's test).
(PDF)

Table S1 **Relative abundance of terminal restriction fragments produced by HaeIII.**
(XLS)

Table S2 **Concentrations of chemical parameters.**
(XLS)

Table S3 **Effects of individual chemical variables on variation in bacterial communities.**
(DOC)

Table S4 **Correlations between chemical variables.**
(XLS)

Table S5 ***A priori*** **groups analysed with generalized discriminant analysis.**
(XLS)

Table S6 **Resulting 14 series of binary model matrices in a particular distance class.**
(DOC)

## References

[1] Bowman JP, McCuaig RD (2003) Biodiversity, community structural shifts, and biogeography of prokaryotes within Antarctic continental shelf sediment. Appl Environ Microbiol 69: 2463–2483. 12732511 10.1128/AEM.69.5.2463-2483.2003 PMC154503

[2] Wu L, Kellogg L, Devol AH, Tiedje JM, Zhou J (2008) Microarray-based characterization of microbial community functional structure and heterogeneity in marine sediments from the Gulf of Mexico. Appl Environ Microbiol 74: 4516–4529. 18515485 10.1128/AEM.02751-07 PMC2493184

[3] Berglund BE, Sandgren P, Barnekow L, Hannon G, Jiang H, et al (2005) Early Holocene history of the Baltic Sea, as reflected in coastal sediments in Blekinge, southeastern Sweden. Quat Int 130: 111–139.

[4] Zillen L, Conley DJ, Andrén T, Andrén E, Björck S (2008) Past occurrences of hypoxia in the Baltic Sea and the role of climate variability, environmental change and human impact. Earth Sci Rev 91: 77–92.

[5] Andrén E, Andrén T, Kunzendorf H (2000) Holocene history of the Baltic Sea as a background for assessing records of human impact in the sediments of the Gotland basin. Holocene 10: 687–702.

[6] Zheng Y, Weinman B, Cronin T, Fleisher MQ, Anderson RF (2003) A rapid procedure for the determination of thorium, uranium, cadmium and molybdenum in small sediment samples by inductively coupled plasma-mass spectrometry: Application in Chesapeake Bay. Appl Geochem 18: 539–549.

[7] Sohlenius G, Westman P (1998) Salinity and redox alternations in the northwestern Baltic Proper during the late Holocene. Boreas 27: 101–114.

[8] Emeis K-C, Struck U, Blanz T, Kohly A, Voß M (2003) Salinity changes in the central Baltic Sea (NW Europe) over the last 10000 years. Holocene 13: 411–421.

[9] Widerlund A, Anderson PS (2011) Late Holocene freshening of the Baltic Sea derived from high-resolution strontium isotope analyses of mollusk shells. Geology 39: 187–190.

[10] Brenner WW (2005) Holocene environmental history of the Gotland basin (Baltic Sea) – a micropalaeontological model. Palaeogeogr Palaeoclimateol Palaeoecol 220: 227–241.

[11] Lopez-Buendia AM, Bastida J, Querol X, Whateley MKG (1999) Geochemical data as indicators of palaeosalinity in coastal organic-rich sediments. Chem Geol 157: 235–254.

[12] Miller U (1986) Ecology and palaeoecology of brackish water diatoms with special reference to the Baltic basin. In: Ricard M, editor. Proceedings of the Eighth International Diatom Symposium. Koenigstein: Koeltz Scientific Books. pp. 601–611.

[13] Kemp AES (1996) Palaeoclimatology and Palaeoceanography from Laminated Sediments. Geol Soc Spec Publ No. 116. p. 258.

[14] Nath BN, Bau M, Rao BR, Rao CM (1997) Trace and rare earth elemental variation in Arabian Sea sediments through a transect across the oxygen minimum zone. Geochim Cosmochim Acta 61: 2375–2388.

[15] Flower RJ (1993) Diatom preservation: experiments and observations on dissolution and breakage in modern and fossil material. Hydrobiologia 269–270: 473–484.

[16] Coolen MJL, Hopmans EC, Rijpstra WIC, Muyzer G, Schouten S, et al (2004) Evolution of the methane cycle in Ace lake (Antarctica) during the Holocene: Response of methanogens and methanotrophs to environmental change. Org Geochem 35: 1151–1167.

[17] Coolen MJL, Talbot HM, Abbas BA, Ward C, Schouten S, et al (2008) Sources for sedimentary bacteriohopanepolyols as revealed by 16S rDNA stratigraphy. Environ Microbiol 10: 1783–1803. 18397311 10.1111/j.1462-2920.2008.01601.x

[18] Inagaki F, Okada H, Tsapin AI, Nealson KH (2005) Research paper: Microbial survival – the paleome: A sedimentary genetic record of past microbial communities. Astrobiology 5: 141–153. 15815165 10.1089/ast.2005.5.141

[19] Sorensen KB, Teske A (2006) Stratified communities of active archaea in deep marine subsurface sediments. Appl Environ Microbiol 72: 4596–4603. 16820449 10.1128/AEM.00562-06 PMC1489303

[20] Aquilina A, Knab NJ, Knittel K, Kaur G, Geissler A, et al (2010) Biomarker indicators for anaerobic oxidizers of methane in brackish-marine sediments with diffusive methane fluxes. Org Geochem 41: 414–426.

[21] Coolen MJL, Saenz JP, Giosan L, Trowbridge NY, Dimitrov P, et al (2009) DNA and lipid molecular stratigraphic records of haptophyte succession in the Black Sea during the Holocene. Earth Pla Sci Lett 284: 610–621.

[22] Miettinen A (2004) Holocene sea-level changes and glacio-isostasy in the Gulf of Finland, Baltic Sea. Quat Int 120: 91–104.

[23] Sandgren P, Subetto DA, Berglund BE, Davydova NN, Savelieva LA (2004) Mid-Holocene Littorina transgressions based on stratigraphic studies in coastal lakes of NW Russia. GFF 126: 363–380.

[24] Corinaldesi C, Barucca M, Luna GM, Dell'Anno A (2011) Preservation, origin and genetic imprint of extracellular DNA in permanently anoxic deep-sea sediments. Mol Ecol 20: 642–654. 21155913 10.1111/j.1365-294X.2010.04958.x

[25] Inagaki F, Nunoura T, Nakagawa S, Teske A, Lever M, et al (2006) Biogeographical distribution and diversity of microbes in methane hydrate-bearing deep marine sediments, on the Pacific Ocean margin. Proc Natl Acad Sci U S A 103: 2815–2820. 16477011 10.1073/pnas.0511033103 PMC1413818

[26] Harrison BK, Zhang H, Berelson W, Orphan VJ (2009) Variations in archaeal and bacterial diversity associated with the sulfate-methane transition zone in continental margin sediments (Santa Barbara basin, California). Appl Environ Microbiol 75: 1487–1499. 19139232 10.1128/AEM.01812-08 PMC2655439

[27] Cohan FM (2001) Bacterial species and speciation. Syst Biol 50: 513–524. 12116650 10.1080/10635150118398

[28] Eronen M (1988) A scrutiny of the Late Quaternary history of the Baltic Sea. In: Winterhalter B, editor. The Baltic Sea. Geological Survey of Finland Special Paper 6. pp. 11–18.

[29] Westman P, Borgendahl J, Bianchi TS, Chen NH (2003) Probable causes for cyanobacterial expansion in the Baltic Sea: Role of anoxia and phosphorus retention. Estuaries 26: 680–689.

[30] Hughen KA, Baillie MGL, Bard E, Beck JW, Bertrand CJH, et al (2004) Marine04 marine radiocarbon age calibration, 0–26 cal kyr BP. Radiocarbon 46: 1059–1086.

[31] Lane DJ, Pace B, Olsen GJ, Stahl DA, Sogin ML, et al (1985) Rapid-determination of 16S ribosomal-RNA sequences for phylogenetic analyses. Proc Natl Acad Sci U S A 82: 6955–6959. 2413450 10.1073/pnas.82.20.6955 PMC391288

[32] Abdo Z, Schüette UME, Bent SJ, Williams CJ, Forney LJ, et al (2006) Statistical methods for characterizing diversity of microbial communities by analysis of terminal restriction fragment length polymorphisms of 16S rRNA genes. Environ Microbiol 8: 929–938. 16623749 10.1111/j.1462-2920.2005.00959.x

[33] Wang Q, Garrity GM, Tiedje JM, Cole JR (2007) Naïve Bayesian classifier for rapid assignment of rRNA sequences into the new bacterial taxonomy. Appl Environ Microbiol 73: 5261–5267. 17586664 10.1128/AEM.00062-07 PMC1950982

[34] Cole JR, Chai B, Farris RJ, Wang Q, Kulam-Syed-Mohideen AS, et al (2007) The ribosomal database project (RDP-II): introducing myRDP space and quality controlled public data. Nucleic Acids Res 35: D169–D172. 17090583 10.1093/nar/gkl889 PMC1669760

[35] McArdle BH, Anderson MJ (2001) Fitting multivariate models to community data: A comment on distance-based redundancy analysis. Ecology 82: 290–297.

[36] Anderson MJ (2003) DISTLM forward: a FORTRAN computer program to calculate a distance-based multivariate analysis for a linear model using forward selection. Department of Statistics, University of Auckland, New Zealand. Available: http://www.stat.auckland.ac.nz/,mja/prog/DISTLM_forward_UserNotes.pdf. Accessed 2008 November 27.

[37] Anderson MJ, Willis TJ (2003) Canonical analysis of principal coordinates: A useful method of constrained ordination for ecology. Ecology 84: 511–525.

[38] Oksanen J, Kindt R, Legendre P, O'Hara B, Simpson GL, et al. (2008) Vegan: Community Ecology Package. R package version 1.13-1. Available: http://vegan.r-forge.r-project.org/. Accessed 2011 March 25.

[39] Goslee SC, Urban DL (2007) The ecodist package for dissimilarity-based analysis of ecological data. J Stat Softw 22: 1–19.

[40] R Development Core Team. (2007) R: A language and environment for statistical computing. R Foundation for Statistical Computing, Vienna, Austria. ISBN 3-900051-07-0, URL http://www.R-project.org.

[41] Anderson MJ, Robinson J (2003) Generalized discriminant analysis based on distances. Aust Nz J Stat 45: 301–318.

[42] Kindt R, Coe R (2005) Tree diversity analysis. A manual and software for common statistical methods for ecological and biodiversity studies. World Agroforestry Centre (ICRAF), Nairobi. ISBN 92-9059-179-X.

[43] Anderson MJ (2006) Distance-based tests for homogeneity of multivariate dispersions. Biometrics 62: 245–253. 16542252 10.1111/j.1541-0420.2005.00440.x

[44] Anderson MJ, Gribble NA (1998) Partitioning the variation among spatial, temporal and environmental components in a multivariate data set. Aust J Ecol 23: 158–167.

[45] Bocard D, Legendre P, Drapeau P (1992) Partialling out the spatial component of ecological variation. Ecology 73: 1045–1055.

[46] Legendre P, Legendre L (1998) Numerical ecology: Developments in Environmental Modelling 20. Amsterdam: Elsevier Science B.V.. 853 p.

[47] Anderson MJ (2001) A new method for non-parametric multivariate analysis of variance. Austral Ecol 26: 32–46.

[48] Anderson MJ (2004) DISTLM v.5: a FORTRAN computer program to calculate a distance-based multivariate analysis for a linear model. Department of Statistics, University of Auckland, New Zealand. Available: http://www.stat.auckland.ac.nz/,mja/prog/DISTLM_UserNotes.pdf. Accessed 2008 November 27.

[49] DeSantis TZ, Hugenholtz P, Keller K, Brodie EL, Larsen N, et al (2006) NAST: a multiple sequence alignment server for comparative analysis of 16S rRNA genes. Nucleic Acids Res 34: W394–W399. 16845035 10.1093/nar/gkl244 PMC1538769

[50] Felsenstein J (2005) PHYLIP (phylogeny inference package) version 3.6. Department of Genome Sciences, University of Washington, Seattle.

[51] Letunic I, Bork P (2007) Interactive tree of life (iTOL): An online tool for phylogenetic tree display and annotation. Bioinformatics 23: 127–128. 17050570 10.1093/bioinformatics/btl529

