# Supplementary figures and images for: Sediment Bacterial Communities Reflect the History of a Sea Basin
Source: PLoS One. 2013 Jan 23;8(1):e54326. doi: 10.1371/journal.pone.0054326 (PMC3553170; doi:10.1371/journal.pone.0054326)

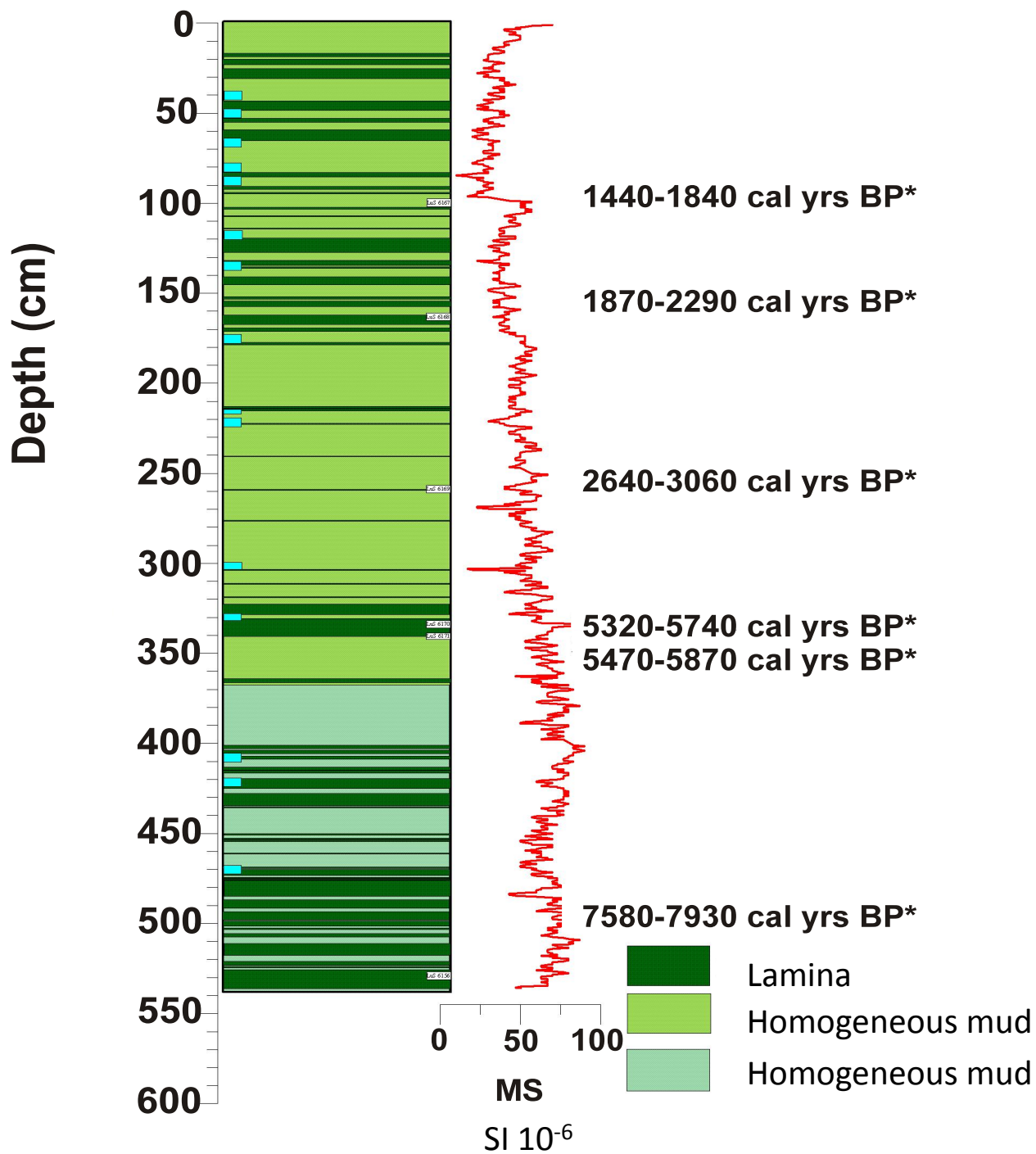

Supplement: Figure S1 — Sediment core taken from the northern Baltic Sea. Two homogeneous mud units contain both homogeneous and bioturbated layers. (PDF) [file pone.0054326.s001.pdf]

Chromium

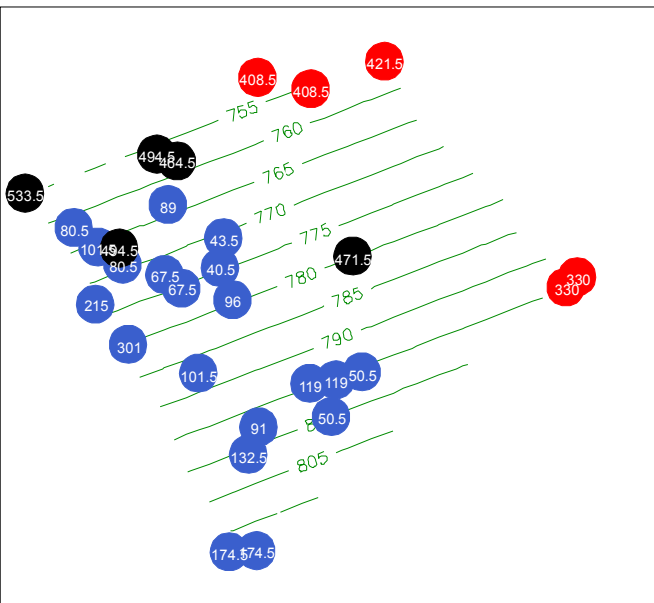

Lead

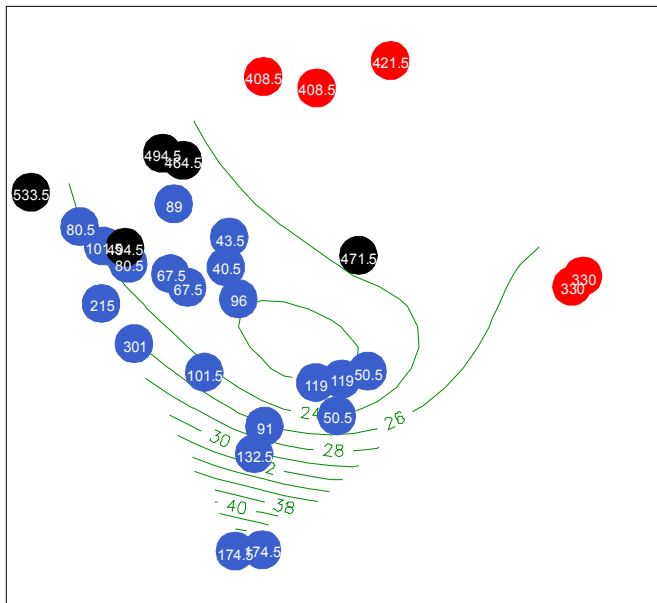

Sodium

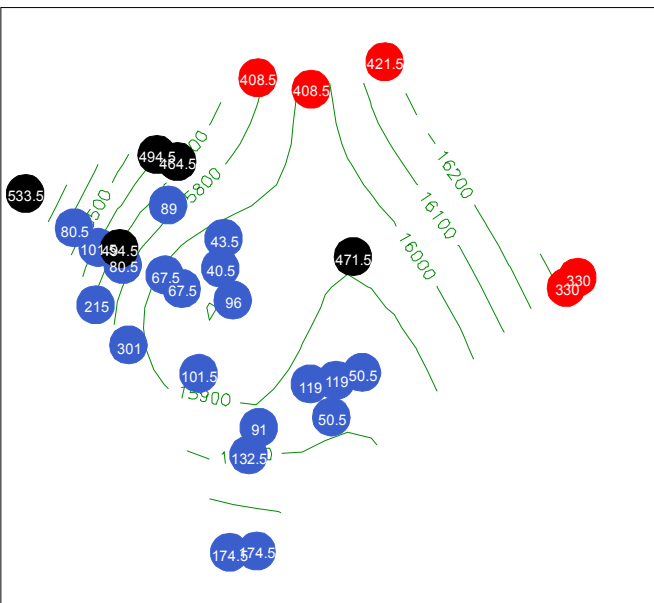

Phosphorus

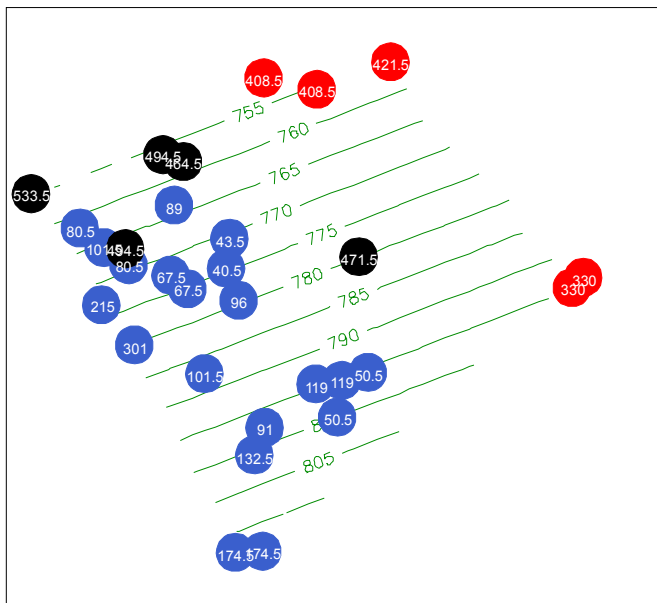

Strontium

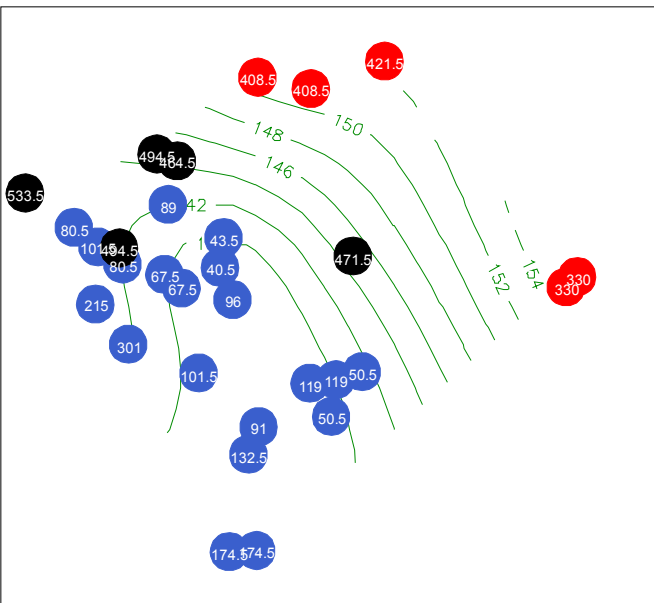

Uranium

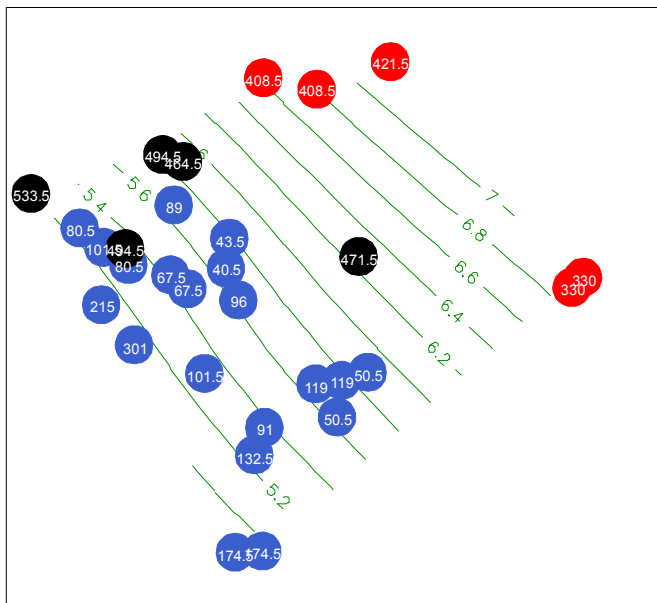

Supplement: Figure S2 — Relationship between chromium, lead, sodium, phosphorus, strontium, uranium and bacterial communities. The chemical variables were fitted on the final bacterial ordination surface. The numbers in the coloured filled circles indicate the subsurface depth of a particular sample (bacterial communities determined by T-RFLP). The green numbers indicate the concentration of a particular chemical parameter. Final model: refer to Figure 1. (PDF) [file pone.0054326.s002.pdf]

Aluminium

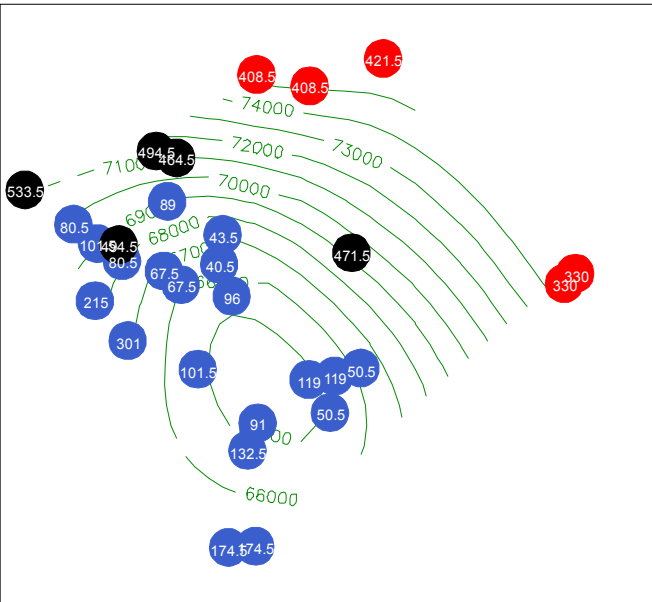

Barium

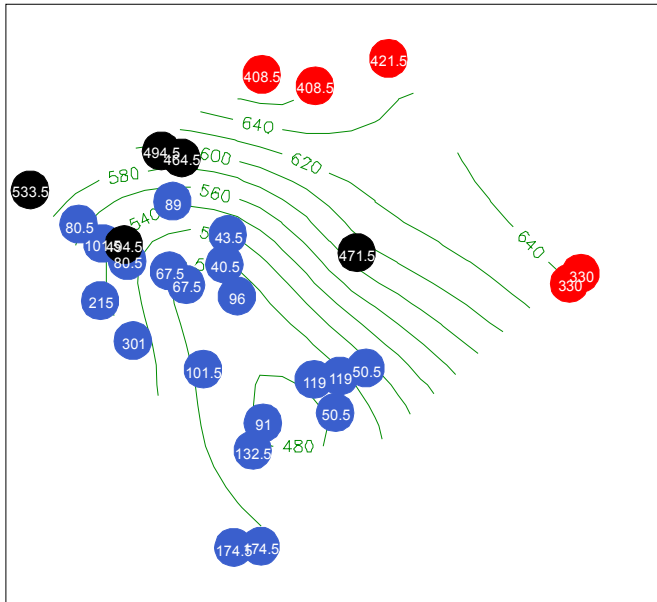

Calcium

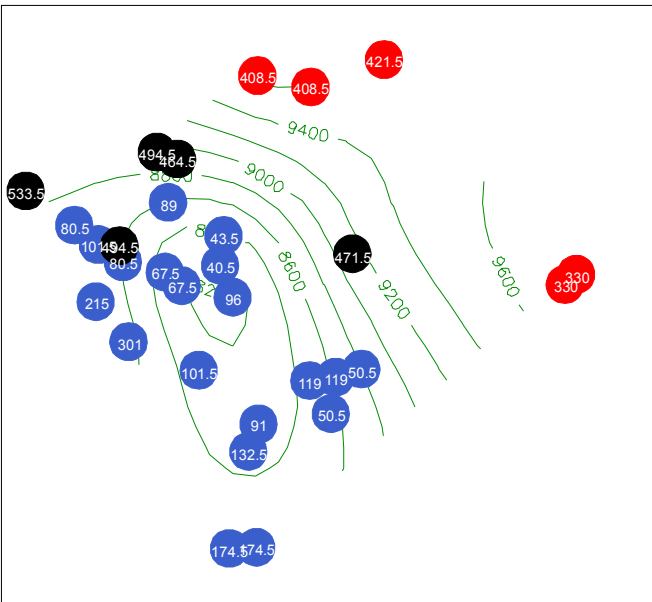

Carbon

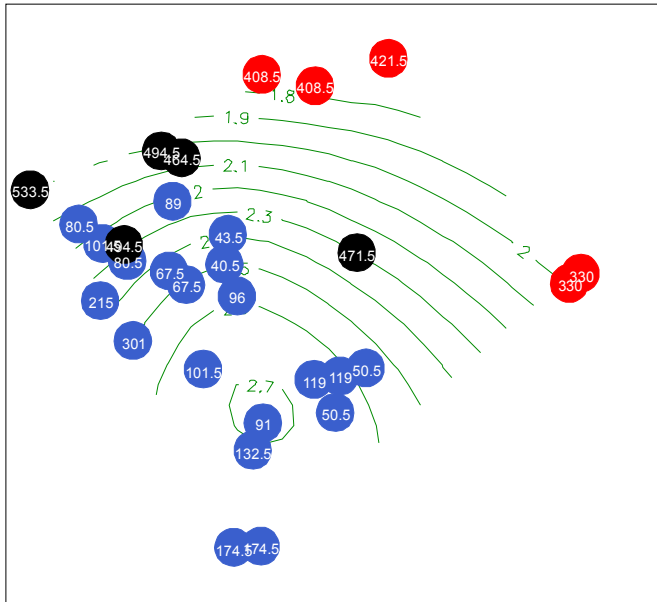

Cobalt

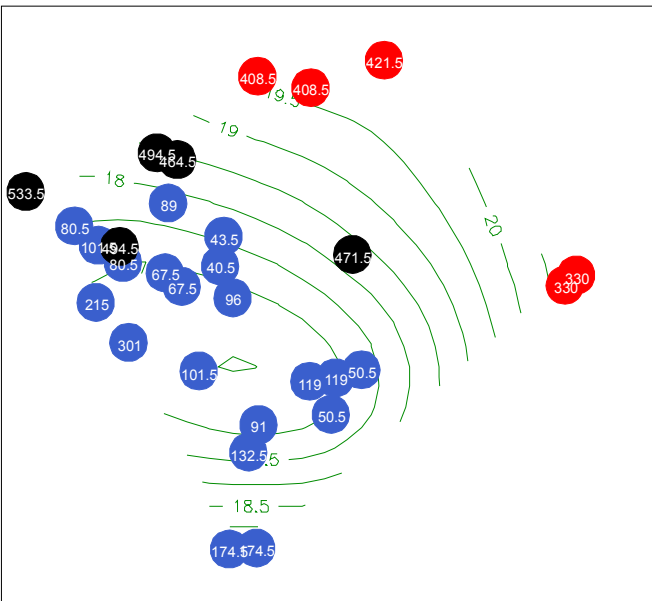

Iron

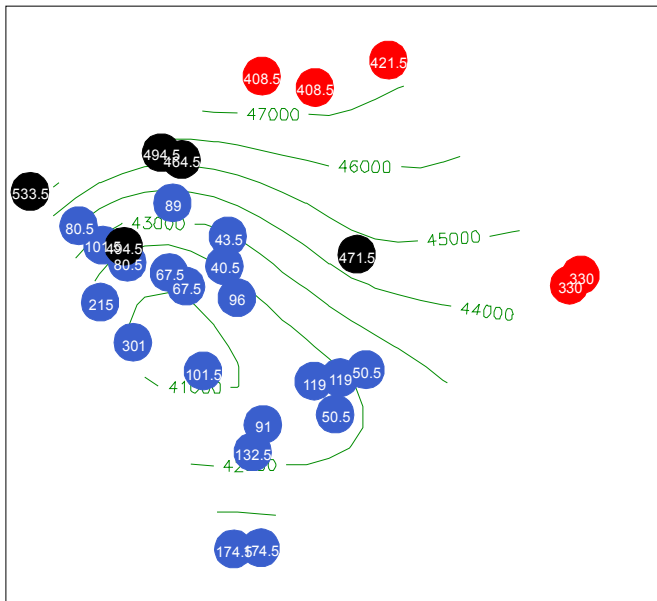

Supplement: Figure S3 — Non linear relationship between aluminium, barium, calcium, carbon, cobalt, iron and bacterial communities. The chemical variables were fitted on the final bacterial ordination surface. The numbers in the coloured filled circles indicate the subsurface depth of a particular sample (the bacterial communities determined by T-RFLP). The green numbers indicate the concentration of a particular chemical parameter. Final model: refer to Figure 1. (PDF) [file pone.0054326.s003.pdf]

# Magnesium

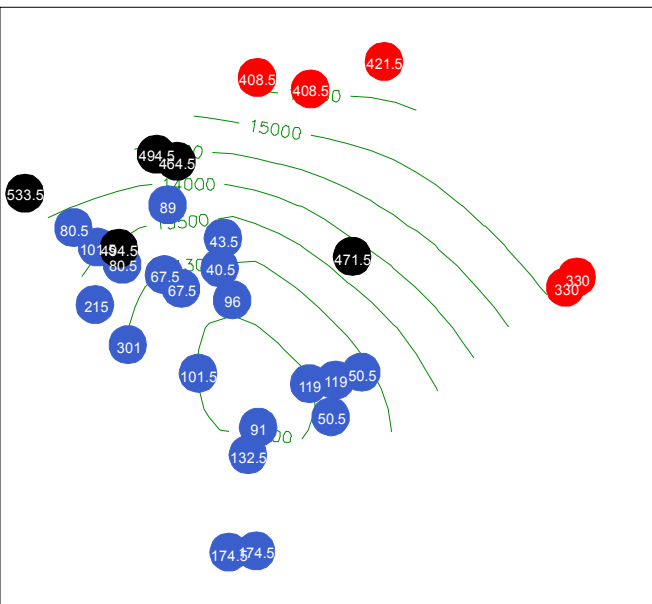

## Nitrogen

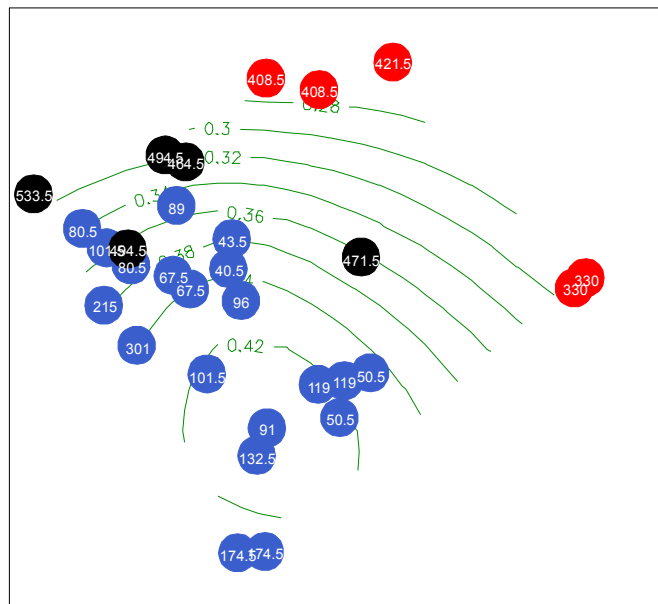

## Thallium

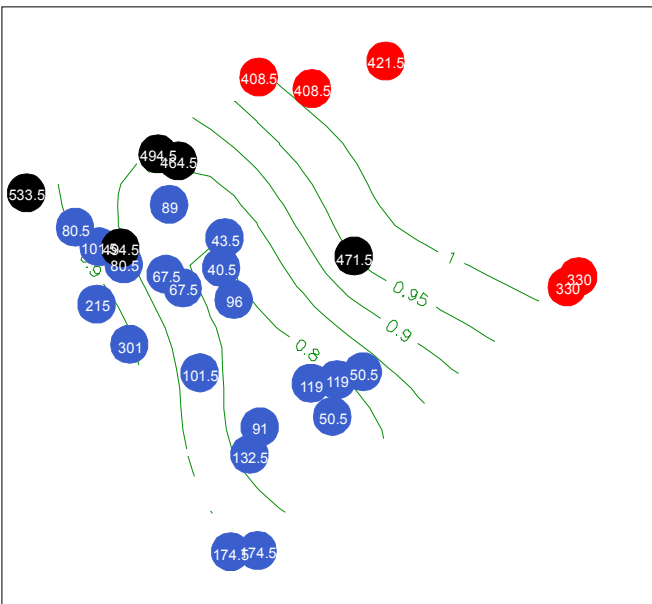

## Vanadium

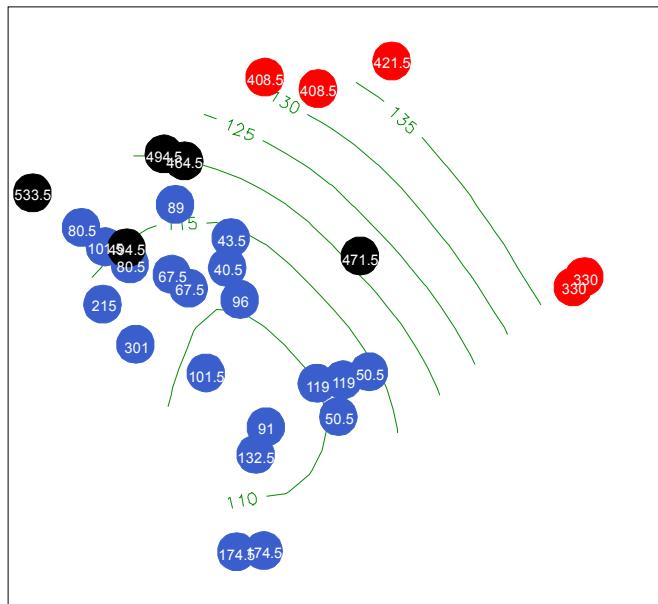

Supplement: Figure S4 — Non linear relationship between magnesium, nitrogen, thallium, vanadium and bacterial communities. Chemical variables were fitted on the final bacterial ordination surface. The numbers on the coloured filled circles indicate the subsurface depth of a particular sample (the bacterial communities determined by T-RFLP). The green numbers indicate the concentration of a particular chemical parameter. Final model: refer to Figure 1. (PDF) [file pone.0054326.s004.pdf]

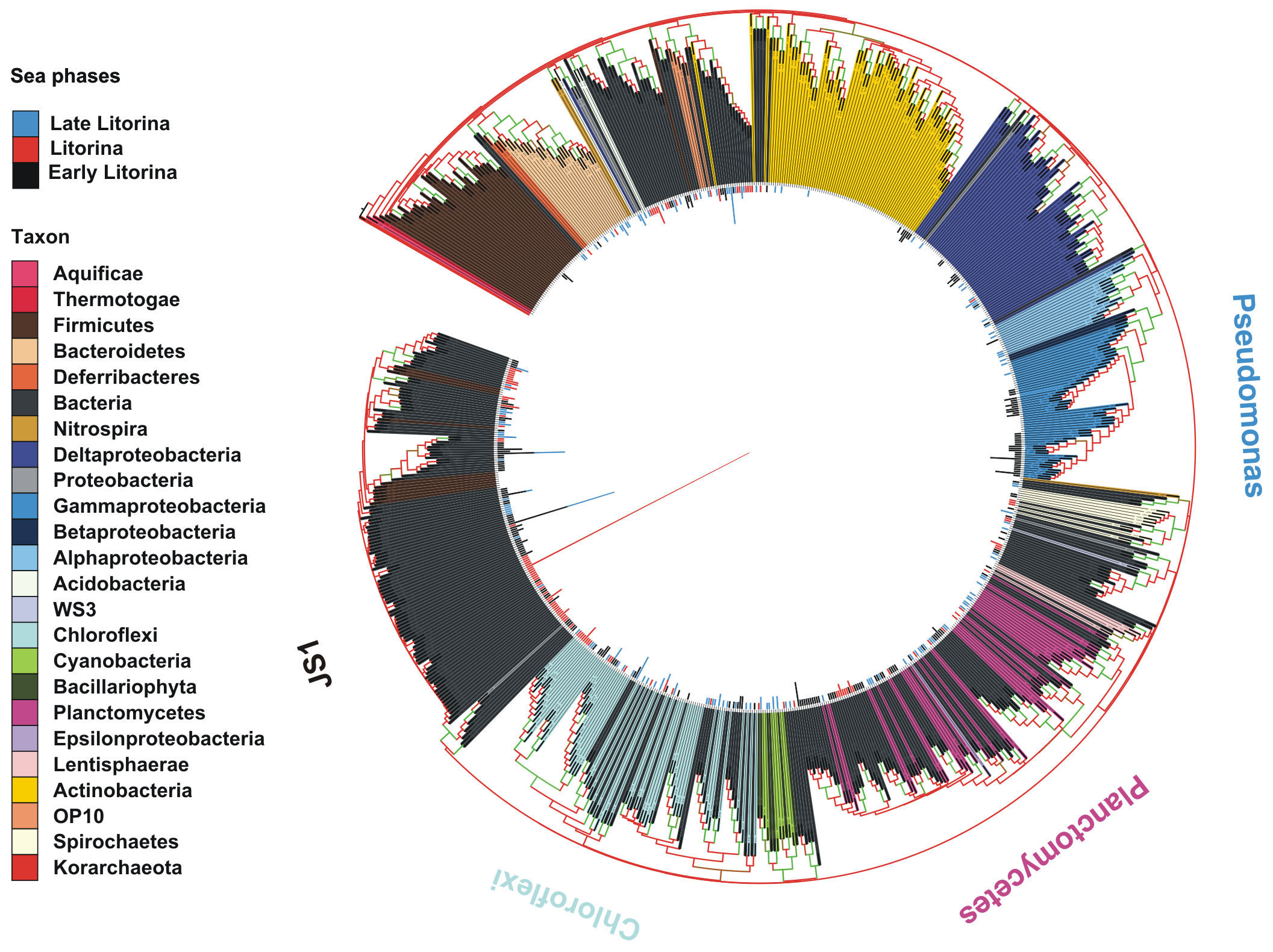

Supplement: Figure S5 — Bootstrap tree of the partial 16S rRNA genes from stratified Baltic Sea sediments. In the inverted circular tree with a stacked bar chart, the red bars indicate the abundance of a leaf (sequence) found in the Litorina Sea laminae (depths 330 and 422 cm), blue bars the abundance of a leaf found in the Late Litorina Sea laminae (depths 91 and 101 cm) and black bars the abundance of a leaf found in the Early Litorina lamina (depth 534 cm). The leaves without bars are reference sequences obtained, using the NAST tool. The clone sequences were assigned to phylum or class level (Proteobacteria) or to genus level (Pseudomonas) by the RDP classifier with an 80% threshold. The putative JS1 clones were assigned, based on the closest sequence matches, using the RDP seqmatch tool. The tree is based on 1000 bootstraps. Those branches with higher than 80% bootstrap values are indicated with red (maximum values) and green (minimum values) colour. (TIF) [file pone.0054326.s005.tif]

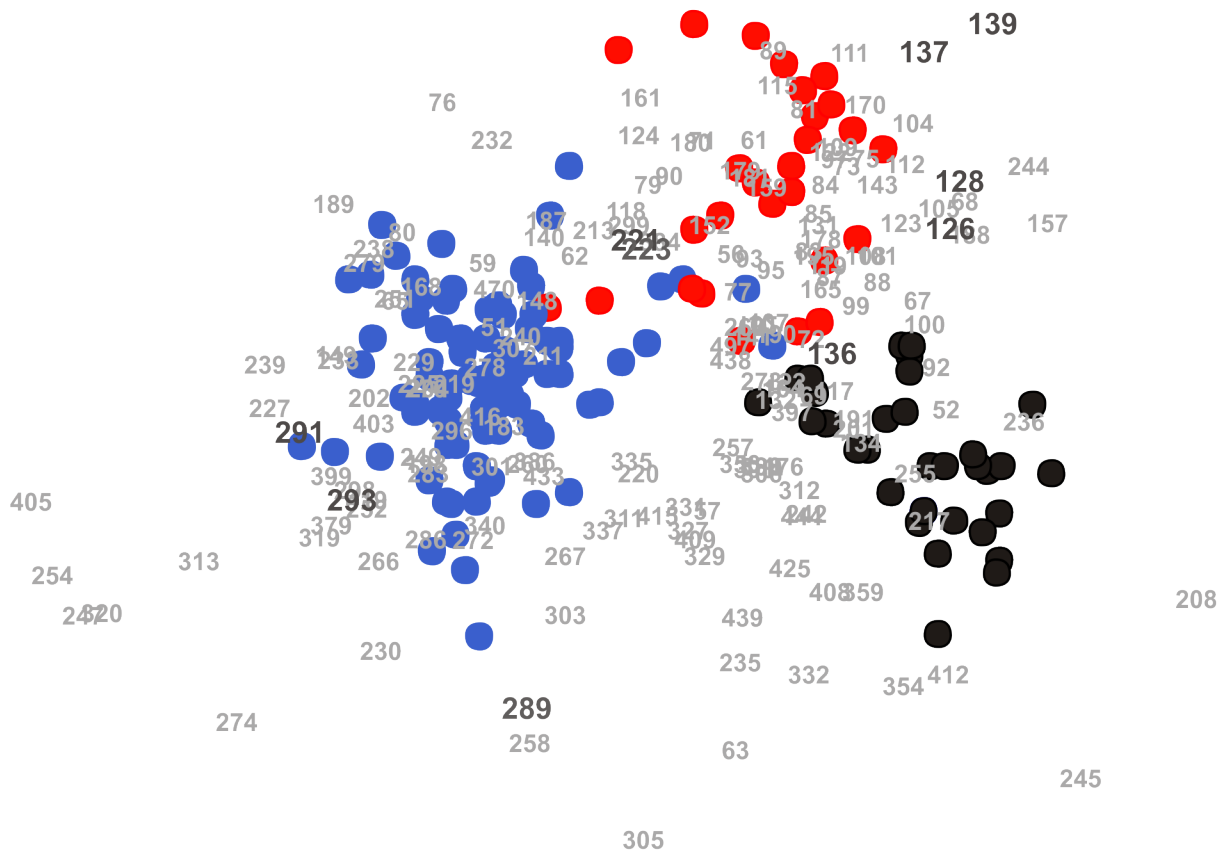

Supplement: Figure S6 — Association between bacterial communities and terminal-restriction fragments from Baltic Sea sediments. The expected/observed (larger font and dark grey) terminal-restriction fragments (T-RFs) of JS1 are 223–225/221, 223; Synechococcus: 128/126, 128 and 137/136, 137, 139; Nodularia: 291/289, 291, 293. The T-RFs expected were determined based on in silico-digested 16S rRNA gene clones. The bacterial communities were determined, based on HaeIII digested 16S rRNA gene clones. A shift of ±2 between the in vitro and in silico T-RFs was allowed. Samples (n = 148) are indicated with the blue (Late Litorina Sea)-, red (Litorina Sea)- and black (Early Litorina Sea)- filled circles. The numbers represent (in bp) (T-RFs, n = 219). Only T-RFs with canonical scores above ±1 for axis 1 and 2 were included. The clone sequences were identified, based on the closest sequence matches, using the RDP seqmatch tool. (PDF) [file pone.0054326.s006.pdf]

Distance to centroid

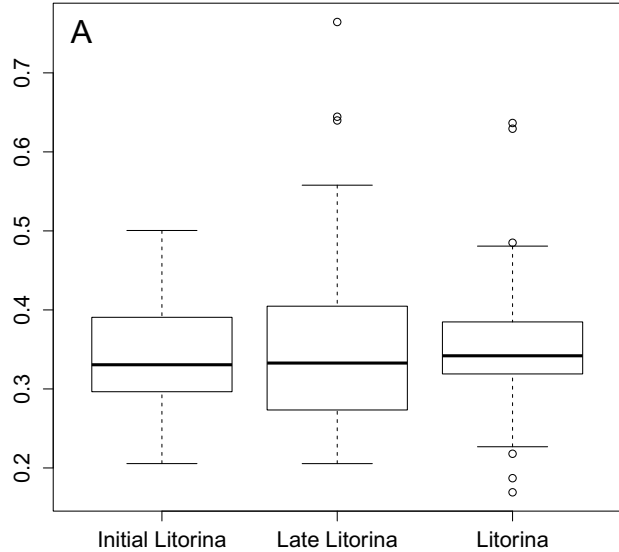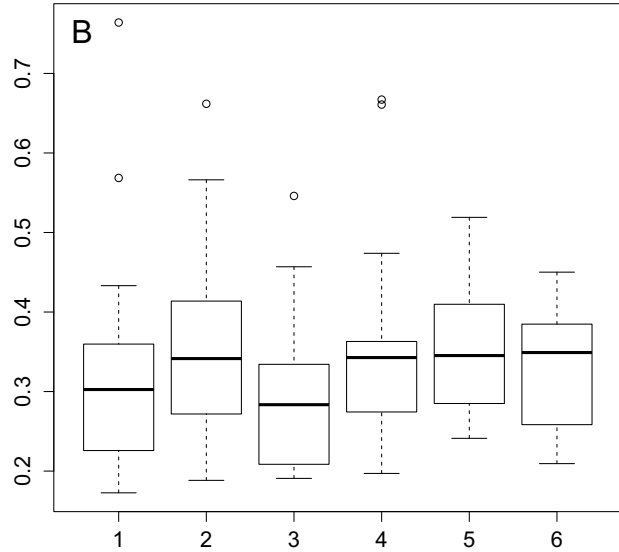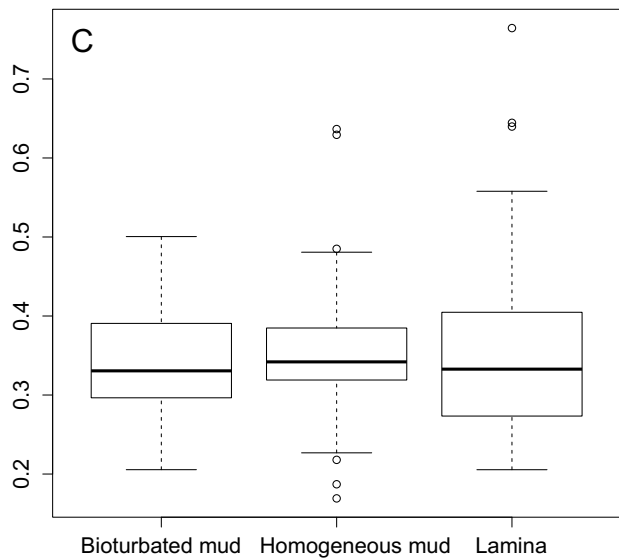

Supplement: Figure S7 — Variance heterogeneities of the 16S rRNA gene terminal-restriction fragment data. (A) Sea phase, (B) depth classes and (C) sediment composition data were calculated by analysis of multivariate homogeneity of group variances (multivariate analogue of Levene's test). (PDF) [file pone.0054326.s007.pdf]
