# Supplementary material for: Sediment Bacterial Communities Reflect the History of a Sea Basin
Source: PLoS One. 2013 Jan 23;8(1):e54326. doi: 10.1371/journal.pone.0054326 (PMC3553170; doi:10.1371/journal.pone.0054326)
Supplement: Table S3 — Effects of individual chemical variables on variation in bacterial communities. (DOC) [file pone.0054326.s010.doc]

**Table S3. Effects of individual chemical variables** on variation in bacterial communities.

| **Variable** | **SS(trace)** | **Pseudo-F** | **P value** | **Prop** |
| --- | --- | --- | --- | --- |
| depth (cm) | 4298.4528 | 3.0438 | 0.0008 | 0.098 |
| As | 1675.0749 | 1.1123 | 0.3356 | 0.0382 |
| Be | 1231.2125 | 0.8091 | 0.6401 | 0.0281 |
| Bi | 2883.1628 | 1.971 | 0.0339 | 0.0658 |
| Cd | 1580.6767 | 1.0473 | 0.3787 | 0.0361 |
| Co | 2918.563 | 1.997 | 0.0279 | 0.0666 |
| Cr | 3456.8593 | 2.3968 | 0.0079 | 0.0789 |
| Cu | 2572.1435 | 1.7452 | 0.0664 | 0.0587 |
| Mo | 1663.6584 | 1.1045 | 0.3413 | 0.0379 |
| Ni | 3726.0524 | 2.6008 | 0.0042 | 0.085 |
| Pb | 3124.75 | 2.1489 | 0.0308 | 0.0713 |
| Rb | 3187.8918 | 2.1957 | 0.0152 | 0.0727 |
| Sb | 914.186 | 0.5963 | 0.8173 | 0.0209 |
| Th | 2938.0991 | 2.0113 | 0.0256 | 0.067 |
| Tl | 3226.8013 | 2.2246 | 0.0121 | 0.0736 |
| U | 3596.7609 | 2.5025 | 0.0062 | 0.082 |
| V | 3565.4896 | 2.4788 | 0.0066 | 0.0813 |
| Zn | 3121.7653 | 2.1467 | 0.0168 | 0.0712 |
| Al | 3552.7231 | 2.4691 | 0.0054 | 0.081 |
| Ba | 4011.386 | 2.82 | 0.002 | 0.0915 |
| Ca | 3153.7287 | 2.1703 | 0.0168 | 0.0719 |
| Fe | 3532.6062 | 2.4539 | 0.0068 | 0.0806 |
| K | 3209.8402 | 2.212 | 0.0136 | 0.0732 |
| Li | 3766.3277 | 2.6315 | 0.0036 | 0.0859 |
| Mg | 3830.8259 | 2.6809 | 0.003 | 0.0874 |
| Mn | 2287.9052 | 1.5417 | 0.1174 | 0.0522 |
| Na | 3476.0275 | 2.4112 | 0.01 | 0.0793 |
| P | 4822.5966 | 3.4608 | 0.0001 | 0.11 |
| S | 1250.4383 | 0.8221 | 0.6046 | 0.0285 |
| Sr | 3417.1376 | 2.3669 | 0.0101 | 0.0779 |
| Ti | 3536.9289 | 2.4572 | 0.0053 | 0.0807 |
| C | 3044.2524 | 2.0894 | 0.0206 | 0.0694 |
| N | 3521.2454 | 2.4454 | 0.0058 | 0.0803 |
