# Supplementary material for: Sediment Bacterial Communities Reflect the History of a Sea Basin
Source: PLoS One. 2013 Jan 23;8(1):e54326. doi: 10.1371/journal.pone.0054326 (PMC3553170; doi:10.1371/journal.pone.0054326)
Supplement: Table S6 — Resulting 14 series of binary model matrices in a particular distance class. (DOC) [file pone.0054326.s013.doc]

**Table S6.** Resulting 14 series of binary model matrices in a particular distance class.

| **$mgram**^a^ | **lag**^b^ | **ngroups**^c^ | **mantelr**^d^ | **p value** |
| --- | --- | --- | --- | --- |
| [1,] | 18.40179 | 1445 | 0.36321006 | 0.00010001 |
| [2,] | 55.20536 | 1326 | 0.24432714 | 0.00010001 |
| [3,] | 92.00893 | 1206 | 0.14777175 | 0.00010001 |
| [4,] | 128.8125 | 1055 | 0.06578783 | 0.03030303 |
| [5,] | 165.61607 | 899 | -0.05369874 | 0.09820982 |
| [6,] | 202.41964 | 820 | -0.07221221 | 0.03790379 |
| [7,] | 239.22321 | 769 | -0.20371501 | 0.00010001 |
| [8,] | 276.02679 | 726 | -0.25990798 | 0.00010001 |
| [9,] | 312.83036 | 693 | -0.2847793 | 0.00010001 |
| [10,] | 349.63393 | 620 | -0.25096751 | 0.00010001 |
| [11,] | 386.4375 | 507 | -0.25987158 | 0.00010001 |
| [12,] | 423.24107 | 423 | -0.13162416 | 0.00890089 |
| [13,] | 460.04464 | 271 | -0.33479232 | 0.00010001 |
| [14,] | 496.84821 | 90 | -0.49344885 | 0.00010001 |

Data matrix was based on samples (n=148) x T-RFs n=219).

^a^Appropriate number of distant classes calculated using Sturge’s rule

^b^Midpoint of the distant class

^c^Number of distances in the class

^d^Mantel r value
